# S163. FEASIBILITY STUDY: MEASURES OF SLEEP AND PHYSICAL ACTIVITY IN PEOPLE WITH SCHIZOPHRENIA

**DOI:** 10.1093/schbul/sby018.950

**Published:** 2018-04-01

**Authors:** Alexandra Berry, Richard Drake, Roger Webb, Darren Ashcroft, Matthew Carr, Alison Yung

**Affiliations:** University of Manchester

## Abstract

**Background:**

People with schizophrenia and related psychotic illnesses have poor physical health and are at an increased risk of developing long-term physical health conditions such as diabetes and heart disease. While this may be due to unhealthy lifestyles, such as lack of physical activity, circadian rhythm problems may also play a part. It is therefore important to be able to measure physical activity and sleep patterns in schizophrenia. This study aims to assess for feasibility by comparing ActiGraph accelerometer data, mobile phone app data and questionnaire data.

**Methods:**

A cross-sectional comparison of different assessment methods of sleep and general activity was used. Assessment methods included:

a) ActiGraph wGT3X-BT accelerometers worn on the waist and wrist for 7 days.

b) Lenovo A Plus smartphone apps ‘SleepBot’ and ‘Google Fit’ installed for the purposes of gathering data on sleep and physical activity patterns for 7 days.

c) Simple Physical Activity Questionnaire taken at baseline and on day 7.

At the seven-day assessment participants were interviewed using a topic guide covering their experiences. This explored the feasibility and acceptability of the measures and possible barriers for implementation.

**Results:**

14 out of a planned 30 participants who met DSM IV-R criteria for schizophrenia spectrum psychoses have been recruited across Greater Manchester from wards and in the community. All participants were retained for the 7-day study duration. Preliminary assessment has shown concordance between the different measures. 3 out of the 14 participants engaged in vigorous physical activity during the 7 days. All 14 participants spent more than 50% of their time sedentary during the 7 days. Participants showed fragmented sleep with a high number of awakenings.

**Discussion:**

Using mobile phones and accelerometers are inexpensive and unobtrusive methods for measuring sleep and physical activity. These measures are feasible and acceptable to people with schizophrenia and are therefore suitable for implementation in routine clinical care. The measures can also be used by service users themselves to enhance their ability to monitor their own physical health. Such self-management and monitoring may encourage goal setting and improve autonomy, which have been found to be associated with increased levels of physical activity.

